# Dataset of traumatic myiasis observed for three dominant screw worm species in North West Pakistan with first report of *Wohlfahrtia magnifica* (Schiner)

**DOI:** 10.1016/j.dib.2016.07.053

**Published:** 2016-08-03

**Authors:** Farrah Zaidi, Syeda Hira Fatima, Ayesha Gul

**Affiliations:** a Department of Zoology, University of Peshawar, Pakistan; b Department of Space Science, Institute of Space Technology, Islamabad, Pakistan

**Keywords:** *Wohlfahrtia magnifica*, Screwworm, Myiasis

## Abstract

Regional surveys were carried out in different parts of North West Pakistan among domestic animals (*N*=57,921) including pets and livestock identifying cases of traumatic myiasis (*n*=1037). A total of four surveys focused general livestock population during Eid ul Adha (Eid surveys; incidence=1.21%) while another four surveys (Miscellaneous surveys; incidence=7.34%) targeted animal population brought to veterinary hospitals and dispensaries. Timeframe spanned four years from 2012 to 2015. Maggots were sampled and location of the wound was recorded for each host. Taxonomic identification used light and electron microscopic techniques. Our dataset shows three species as principle agents of myiasis (*n*=882) including *Chrysomya bezziana* Villeneuve (*n*=394)*, Wohlfahrtia magnifica* (*n*=244) and *Lucilia cuprina* Wiedemann (*n*=244). Others (*n*=155) including *Chrysomya megacephala* (Fabricius)*, Chrysomya rufifacies* (Macquart)*, Lucilia sericata* (Meigen)*, Lucilia illustris* (Meigen)*, Lucilia porphyrina* (Walker)*, Hemipyrellia ligguriens* (Wiedemann)*, Calliphora vicina* (Robineau-Desvoidy)*, Sarcophaga crassipalpalis* (Macquart) and *Sarcophaga species* were identified as species of minor importance. The obligatory screwworm species *W. magnifica* is a first report from Pakistan. The results based on this dataset are presented in a recent publication “Distribution Modeling of three screwworm species in the ecologically diverse landscape of North West Pakistan” (Zaidi et al., 2016) [1].

**Specifications Table**

**Table:** 

Subject area	*Zoology*
More specific subject area	*Veterinary Entomology*
Type of data	*Supplementary data, Figures*
How data was acquired	*Samples were collected through eight regional surveys spanning four years. Target animals included apparently healthy livestock brought for sale during Eid ul Adha and or all animals brought to vet hospitals for examination purposes. Third instar larvae were extracted from wounds using gentle push of a finger, forceps or brush. Wound location was noted and GPS coordinates were recorded for three screwworm species.*
Data format	*Raw, filtered, analyzed*
Experimental factors	*Third instar larvae removed from infested animals*
Experimental features	*Third instar larvae were microscopically examined and reared to identify various species causing myiasis.*
Data source location	*North West Pakistan (Khyber Pakhtunkhwa)*
Data accessibility	*Data is with this article*

**Value of the data**

• Traumatic myiasis is a significant health issue among livestock and human subjects with manifold economic implications. Identification of agents of traumatic myiasis and their spatial distribution in a region can aid in disease prevention.
• This data can be further used to enhance species distribution modeling of both economically and medically important screwworm species.
• Explicit assumption can be made on species range shifts which might further help us to understand changing climatic patterns and their effects on species distribution.
• A thorough and rigorous data to further study screwworm species.

## 1 Data

**Fig. 1:**
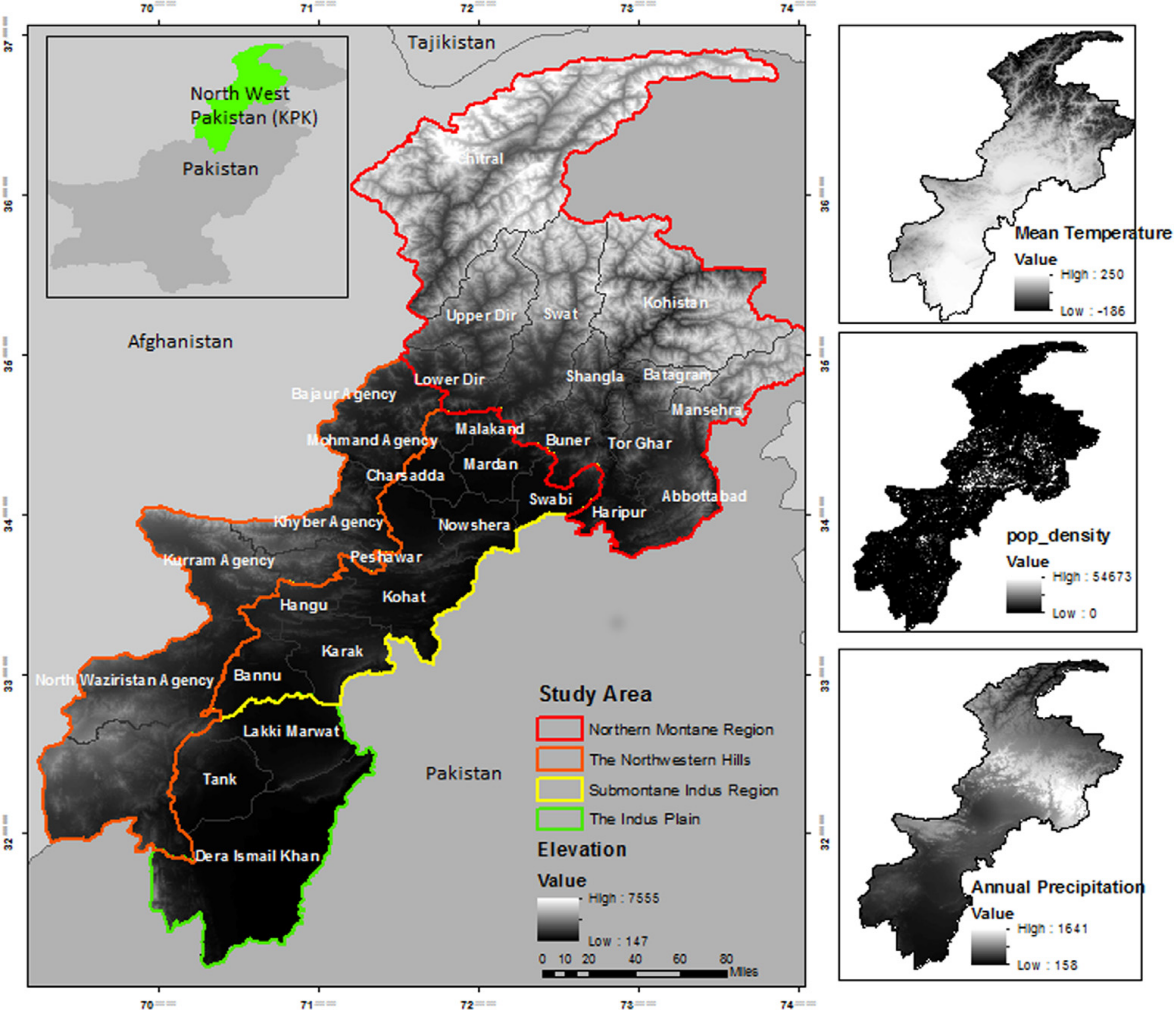
Study Area classified into four eco zones with distinctive environmental features.

**Fig. 2:**
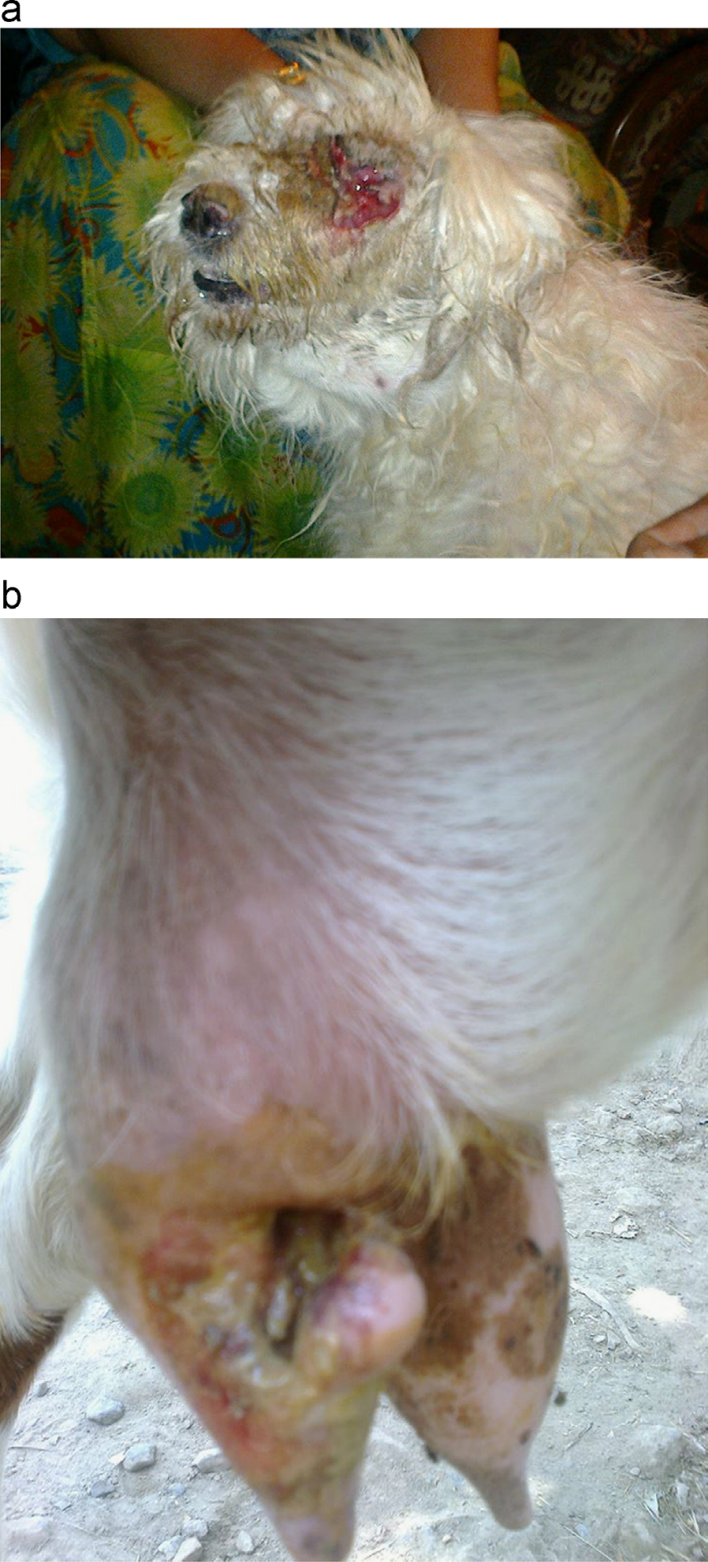
Photographs depicting severity of myiasis among hospital subjects. (a) Dog with ocular myiasis. (b) Maggot infested udder of a goat.

**Fig. 3:**
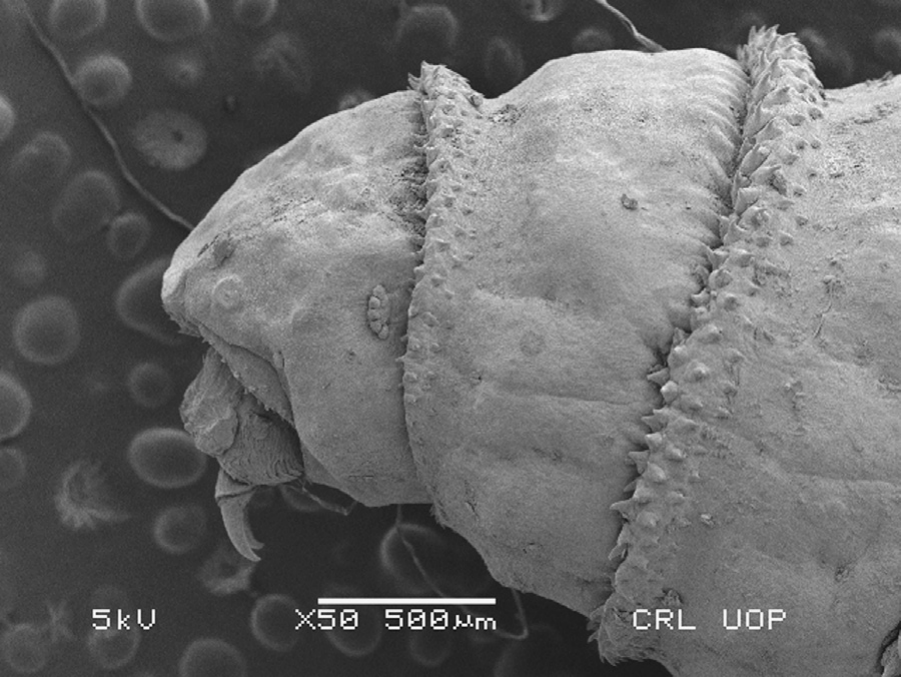
Electron micrograph showing cephalic region of a third instar larva of *Chrysomya bezziana* detailing a five lobed anterior spiracle.

Sampling was performed during eight surveys from general livestock population (Eid Surveys) and or domestic animals brought to veterinary hospitals (Misc. surveys) in North West Pakistan (Fig. 1). Mild cases of myiasis prevailed among Eid livestock; in contrast most severe cases were witnessed among hospital subjects (Fig. 2). Locality of host animals was noted using GPS. Wound location was recorded for each host (Supplementary Tables 1–3). Maggots were identified using light and electron microscopy (Fig. 3). Incidence was calculated for different species for instance.

## 2 Experimental design materials and methods

### 2.1 Study area

North West Pakistan is geographically a dynamic region divided into four eco zones (Fig. 1). Spatial distribution of three species is further discussed in a recent publication [1].

### 2.2 Methodology

The third instar larvae were selected for Scanning Electron Microscopic analysis because of their mature and developed characters. Each specimens was treated with a mixture of glutaraldehyde (2.5%) and phosphate buffer solution (PBS) for primary fixation (pH: 7.4, temperature: 4 °C, treatment duration: 24 h). After following a standard protocol [2] the specimens were ready for electron microscopic examination.
