# Supplementary material for: Dataset of traumatic myiasis observed for three dominant screw worm species in North West Pakistan with first report of Wohlfahrtia magnifica (Schiner)
Source: Data Brief. 2016 Aug 3;8:1333–7. doi: 10.1016/j.dib.2016.07.053 (PMC4990641; doi:10.1016/j.dib.2016.07.053)
Supplement: Supplementary file 1 — Supplementary material [file mmc1.doc]

**Conflict of interest form**

The authors declare that there is no conflict of interest.
